# Enhancement of Anandamide-Mediated Endocannabinoid Signaling Corrects Autism-Related Social Impairment

**DOI:** 10.1089/can.2015.0008

**Published:** 2016-02-01

**Authors:** Don Wei, Drake Dinh, DaYeon Lee, Dandan Li, Allison Anguren, Guillermo Moreno-Sanz, Christine M. Gall, Daniele Piomelli

**Affiliations:** ^1^ Department of Anatomy and Neurobiology, University of California, Irvine, Irvine, California.; ^2^ Department of Ophthalmology, The Fourth Affiliated Hospital of Harbin Medical University, Harbin, China.; ^3^ Department of Biological Chemistry, University of California, Irvine, Irvine, California.; ^4^ Unit of Drug Discovery and Development, Italian Institute of Technology, Genova, Italy.

**Keywords:** autism spectrum disorders, CB_1_, fatty acid amide hydrolase, Fragile X Syndrome, social approach

## Abstract

**Introduction:** We recently uncovered a signaling mechanism by which the endocannabinoid anandamide mediates the action of oxytocin, a neuropeptide that is crucial for social behavior, to control social reward. Oxytocin signaling has been implicated in autism spectrum disorder (ASD), and social reward is a key aspect of social functioning that is thought to be disrupted in ASD. Therefore, as a proof of principle for the core component of ASD—social impairment—we tested an endocannabinoid-enhancing compound on two widely studied mouse models of ASD, the BTBR and *fmr1^−/−^* (model of *Fragile X* Syndrome).

**Methods:** We used the established three-chambered social approach test. We specifically increased the activity of anandamide by administering the compound URB597, a selective inhibitor of fatty acid amide hydrolase (FAAH), the hydrolytic enzyme for anandamide.

**Results:** Remarkably, we found that FAAH blockade completely reversed the social impairment in both mouse models. CB_1_ receptor blockade prevented the prosocial action of FAAH inhibition in BTBR mice. These results were likely independent of effects on anxiety, as FAAH inhibition did not alter the performance of BTBR mice in the elevated plus maze.

**Conclusions:** The results suggest that increasing anandamide activity at CB_1_ receptors improves ASD-related social impairment and identify FAAH as a novel therapeutic target for ASD.

## Introduction

A core feature across autism spectrum disorder (ASD) is impairment in social functioning.^1,2^ People with ASD restrict themselves to repetitive behaviors and show deficits in social reciprocity and communication.^1^ The underlying basis for social impairment in ASD is unknown, and no pharmacological treatment is available. One theory—the social motivation theory—posits that the psychopathology of ASD is rooted in a decreased desire to be social.^3,4^

The neural substrates of normal social behavior are only now beginning to emerge.^5^ Perhaps the best account so far has come from the study of oxytocin. This neuropeptide is crucial in many aspects of social behavior, including affiliation^6^ and reward.^7^ Investigations are ongoing into the contributions of the oxytocin system to ASD and oxytocin-based therapies for ASD.^8^ Recent reports suggest that early treatment with oxytocin may be useful for improving social behavior in animal models^9^ as well as in human patients.^10^ Nevertheless, identifying the key neural systems underlying social behavior and understanding how they interact with oxytocin remain an enormous challenge.^5^

One candidate is the endogenous cannabinoid (“endocannabinoid”) system, a modulatory neurotransmitter system that may play an important role in social behavior. This signaling complex consists of lipid-derived messengers called “endocannabinoids,” whose actions in the brain are mainly, although not exclusively, mediated through CB_1_ cannabinoid receptors. A series of enzymes catalyze endocannabinoid synthesis and degradation to control the activity of these substances.^11,12^ Fatty acid amide hydrolase (FAAH) catalyzes the intracellular hydrolysis of the endocannabinoid anandamide. In an effort aimed at probing anandamide function in social behavior, we found that genetic removal of FAAH in mice increases direct social interactions,^13^ while Trezza et al. noted that pharmacological FAAH inhibition promotes social play in juvenile rats.^14^ More recently, we identified a signaling mechanism by which oxytocin drives anandamide-mediated signaling at CB_1_ receptors to control the rewarding properties of social interactions.^15^ Social contact among male mice stimulates the mobilization of anandamide in the nucleus accumbens (NAc), a key brain region for reward signaling, in an oxytocin-dependent manner. Conversely, in isolated male mice, specifically stimulating oxytocin neurons in the paraventricular nucleus of the hypothalamus drives anandamide mobilization in the NAc. Consequent activation of CB_1_ receptors is necessary and sufficient to regulate social reward. Anandamide enhancement offsets the effects of oxytocin receptor blockade on social reward and NAc activity.^15^ In addition, human studies also support the notion that the endocannabinoid system affects social behavior. Acutely intoxicated marijuana users enjoy interacting more and feel more connected and empathetic.^16,17^ Chakrabarti and Baron-Cohen reported that a polymorphism in the CB_1_ cannabinoid receptor gene modulates social gaze.^18^

Based on those studies, we hypothesized that enhancement of anandamide-mediated signaling at CB_1_ receptors might offer a therapeutic strategy for ASD-related social impairment. As a first test of this idea, we examined the effects of a well-characterized FAAH inhibitor, the compound URB597,^19^ in two established mouse models of ASD: (1) BTBR mice, an inbred strain discovered through the Mouse Phenotype Project, which shows pronounced deficits in social approach, reciprocal social interactions, and juvenile play,^20,21^ and (2) *fmr1^−/−^* mutant mice—a model of *Fragile X* Syndrome, the most common monogenetic cause of ASD, which features persistent social deficits.^22–24^ We show that FAAH inhibition substantially improves social behavior in BTBR and *fmr1^−/−^* mice and that this effect is independent of anxiety modulation.

## Materials and Methods

### Animals

We used male mice (8–10 weeks) bred at UC Irvine. The mice were weaned at P21 and group reared in cages of four to five animals. Testing was done during the light cycle (on at 0630 h and off at 1830 h). Naive mice were used for each behavioral experiment. All procedures met the National Institute of Health guidelines for the care and use of laboratory animals and were approved by the Institutional Animal Care and Use Committee at the University of California, Irvine.

### Drug preparation and treatment

URB597 (synthesized in the laboratory) and AM251 (Cayman Chemicals) were dissolved in a vehicle of saline/propylene glycol/Tween-80 (90/5/5, v/v). URB597 and AM251 were administered i.p. 3 h before starting the behavioral testing.^25^ Drugs were administered at 4 μL/g weight of the animal.

### FAAH assay

Procedures were described previously.^26^ Briefly, reactions were conducted with tissue homogenate dissolved in 0.5 mL Tris buffer containing [^3^H]-anandamide at 37°C for 30 min. Reactions were stopped with 1 mL of chloroform/methanol (1:1, v/v). Samples were centrifuged at 3000 *g* for 10 min at 4°C. Radioactivity in the aqueous layer was measured by liquid scintillation counting.

### Lipid analyses

Procedures were described previously.^27^ Briefly, tissue samples were homogenized in methanol containing internal standards for H^2^-anandamide, H^2^-oleoylethanolamide (H^2^-OEA), and ^2^H_8_-2-arachidonoyl-*sn*-glycerol (^2^H_8_-2-AG) (Cayman Chemicals). Lipids were separated by a modified Folch-Pi method using chloroform/methanol/water (2:1:1) and open-bed silica column chromatography. For liquid chromatography–mass spectrometry (LC/MS) analyses, we used a 1100 liquid chromatography system coupled to a 1946D-mass spectrometer detector equipped with an electrospray ionization interface (Agilent Technologies). The column was a ZORBAX Eclipse XDB-C18 (2.1×100 mm, 1.8 μm; Agilent Technologies). We used a gradient elution method as follows: solvent A consisted of water with 0.1% formic acid, and Solvent B consisted of acetonitrile with 0.1% formic acid. The separation method used a flow rate of 0.3 mL/min. The gradient was 65% B for 15 min, then increased to 100% B in 1 min, and kept at 100% B for 14 min. The column temperature was 15°C. Under these conditions, Na^+^ adducts of anandamide/H^2^-anandamide had retention times (Rt) of 6.9/6.8 min and *m/z* of 348/352, OEA/H^2^-OEA had Rt 12.7/12.6 min and *m/z* 326/330, and 2-AG/^2^H_8_-2-AG had Rt 12.4/12.0 min and *m/z* 401/409. An isotope dilution method was used for quantification.

### Three-chambered social approach task

Previously established methods were followed.^21^ Test mice were habituated to an empty three-chambered acrylic box (40.6×21.6 cm). Habituation included a 10-min session in the center chamber with doors closed and then a 10-min session in all chambers with doors open. Test mice were then tested in a 10-min session. Subjects were offered a choice between a novel object and a novel mouse in opposing side chambers. The novel object was an empty inverted pencil cup and the novel social stimulus mouse was a sex, age, and weight-matched 129/SvImJ mouse. These mice were selected because they are relatively inert, and they were trained to prevent abnormal behaviors, such as biting the cup. Weighted cups were placed on top of the pencil cups to prevent climbing. Low lighting was used—all chambers were measured to be 5 lux before testing. The apparatus was thoroughly cleaned with SCOE 10X odor eliminator (BioFOG) between trials to preclude olfactory confounders. Object/mouse side placement was counterbalanced between trials. Chamber time scoring was automated using image analysis in ImageJ. Sniffing time was scored by trained assistants who were unaware of treatment conditions. Excluded were subjects with outlying inactivity.

### Elevated plus maze

The procedure was based on previously published methods.^28^ The maze was made of black Plexiglas and consisted of two open arms (30×5 cm) and closed arms (30×5 cm, with 15-cm side panels on closed arms). The arms extended from a center square (5×5 cm). The maze was mounted on a Plexiglas base and raised 39 cm above the ground. Lighting consisted of two 40 W incandescent bulbs, each hanging at a height of 1 m above the open arms of the test apparatus. The floor of the apparatus was cleaned with a SCOE 10X odor eliminator (BioFOG) between trials. The animals were placed in the center square and allowed to freely explore for 5 min. The amount of time spent in each arm and the number of entries into each arm were quantified by EthoVision 3.1 video tracking system (Noldus).

### Statistical analyses

Results are expressed as mean±SEM. Significance was determined by the two-tailed Student's *t*-test and one-way or two-way analysis of variance (ANOVA) with Tukey's *post hoc* test. Differences were considered significant if *p*<0.05. Analyses were conducted using GraphPad Prism (GraphPad Software).

## Results

### FAAH inhibition corrects social impairment in BTBR mice

**FIG. 1.**
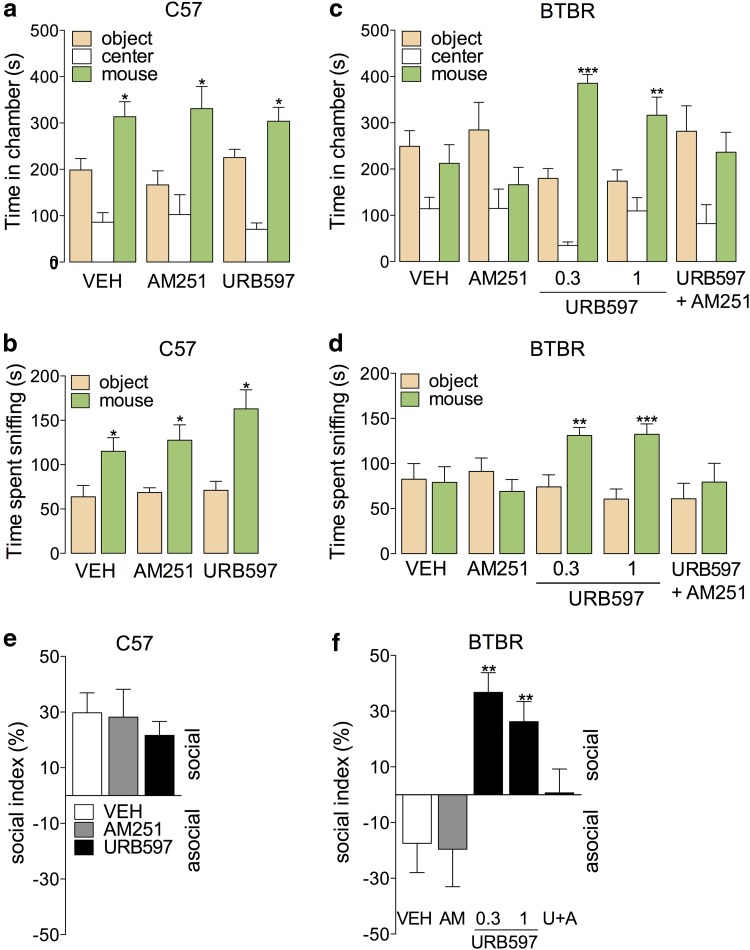
Anandamide activity at CB_1_ receptors corrects social approach impairment in BTBR mice. **(a, b)** Time spent in chamber **(a)** or sniffing **(b)** by C57Bl6J mice in the three-chambered social approach assay after treatment with the CB_1_ inverse agonist AM251 (2 mg kg^−1^, i.p.) or the FAAH inhibitor URB597 (1 mg kg^*−*1^, i.p.). **(c, d)** Chamber and sniffing times of BTBR mice treated with AM251 (2 mg kg^−1^, i.p.), URB597 (i.p.), or AM251 and URB597 in concert. **(e, f)** Social index, calculated as (social chamber time–object chamber time)/(total side-chamber time). Results are expressed as mean±SEM. **(a–f)** *n*=12–16. **(a–d)** Student's *t*-test, comparing object to social, or VEH to URB597; **(e, f)** one-way ANOVA with Tukey's *post hoc* test, comparing to VEH. *p<0.05, **p<0.01, ***p<0.001. ANOVA, analysis of variance; FAAH, fatty acid amide hydrolase.

We used the CB_1_-receptor inverse agonist, AM251, or the FAAH inhibitor, URB597,^25^ to probe the role of anandamide in social behavior of young adult mice. Social activity was evaluated in the widely used social approach test.^20,21^ The mice were placed in a dimly lit three-chambered apparatus and given a choice between (1) a novel inert mouse restrained by an inverted pencil cup in one chamber or (2) a novel object (empty pencil cup) in the opposite chamber.^20^ We first evaluated maximally effective doses of AM251 (2 mg kg^−1^, i.p.)^29^ or URB597 (1 mg kg^−1^, i.p.)^25^ in socially normal C57Bl6J mice and found that neither drug altered the two outcome measures of the test, namely the time spent in the social chamber and the time spent sniffing the target mouse (Fig. 1a, b).

In contrast to C57Bl6J mice, BTBR mice show no social preference in the test (Fig. 1c, d).^20,21^ However, administration of URB597 (0.3 or 1 mg kg^−1^, i.p., 3 h before testing) significantly increased the time BTBR mice spent in the social chamber and sniffing, to levels that were comparable to those displayed by control, socially normal C57Bl6J mice (Fig. 1c, d). The effect of FAAH inhibition on social approach depended on CB_1_ receptors and, thus, presumably on anandamide accumulation, because it was prevented by concomitant administration of AM251 (2 mg kg^−1^, i.p.) (Fig. 1c, d). The results on time spent in the social chamber are summarized as an index (Fig. 1e, f).

**FIG. 2.**
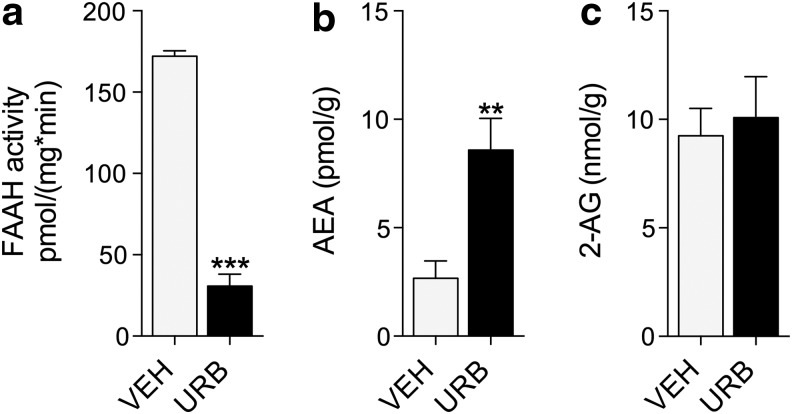
URB597 inhibits FAAH and elevates anandamide levels in the forebrain of BTBR mice. **(a)** FAAH enzymatic activity, **(b)** anandamide (AEA) levels, and **(c)** 2-arachidonoyl-*sn*-glycerol (2-AG) levels after treatment with URB597 (1 mg kg^−1^, i.p.). **p<0.01; ***p<0.001; ANOVA.

Using an enzymatic activity assay and LC/MS, we confirmed that URB597 inhibited FAAH and substantially increased the levels of anandamide in the forebrain of BTBR mice (Fig. 2a, b), without affecting levels of the other endocannabinoid 2-arachidonoyl-*sn*-glycerol (2-AG) (Fig. 2c). Together, the results suggest that elevated anandamide activity at CB_1_ receptors corrects social approach behavior in BTBR mice, whereas it does not alter social approach in control C57Bl6J mice.

### FAAH inhibition does not alter performance of BTBR mice in the elevated plus maze

**FIG. 3.**
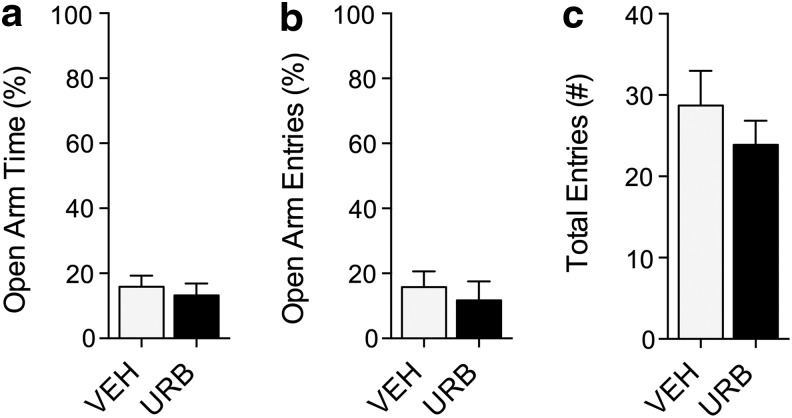
Effects of URB597 in BTBR mice on elevated plus maze under dim lighting conditions. **(a–c)** Open arm time, entries, and total entries in elevated plus maze after treatment with URB597 (1 mg kg^−1^, i.p.). Results are expressed as mean±SEM. *n*=10 per group. Student's *t-*test.

The endocannabinoids are important modulators of stress reactivity in humans and experimental animals.^19,30^ In rodent experiments, this modulation depends on the adverseness of environmental conditions, such as the intensity of ambient lighting.^13,31,32^ To test whether the prosocial actions of FAAH inhibition in BTBR mice might be due to a general reduction in anxiety, we asked whether URB597 exerted anxiolytic-like effects in the elevated plus maze test under the same dim lighting conditions used for the social approach test (5 lux). We found that URB597 (1 mg kg^−1^, i.p.) administered to BTBR mice did not alter the time spent in the open arms, or the number of open-arm entries (Fig. 3a–c), suggesting that the prosocial effect of this compound cannot be attributed to reduced anxiety.

### FAAH inhibition corrects social impairment in *fmr1^−/−^* mice

**FIG. 4.**
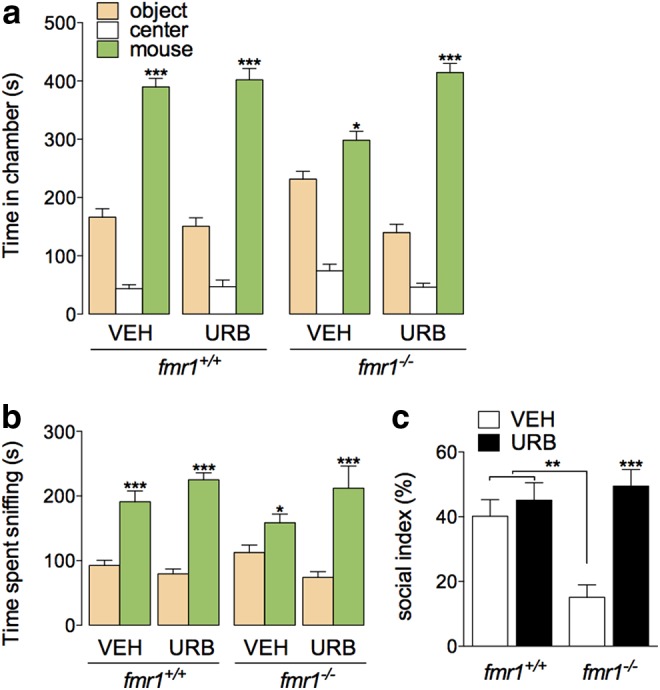
Increasing anandamide activity through FAAH inhibition corrects social approach impairment in *fmr1^−/−^* mice. **(a, b)** Chamber and sniffing times of wild-type (FVB/NJ) or *fmr1^−/−^* mice in the three-chambered social approach assay after treatment with the FAAH inhibitor URB597 (0.3 mg kg^−1^, i.p.). **(c)** Social index, calculated as (social chamber time—object chamber time)/(total side-chamber time). Results are expressed as mean±SEM. *n*=12–14 per group. **(a, b)** Student's unpaired *t*-test, comparing object to social, **(c)** two-way ANOVA with Tukey's *post hoc* test. *p<0.05, **p<0.01, ***p<0.001.

Interpretation of data obtained with BTBR mice is limited by the possibly polygenetic contributions to the phenotype of this inbred strain. Therefore, we asked whether the prosocial effect of anandamide is translatable to a monogenetic model of ASD-related social impairment. *Fmr1^−/−^* mutant mice bred on an FVB/NJ background have been reported to exhibit a deficit in social approach.^22^ In our hands, however, this deficit was not statistically significant (Fig. 4a, b). Nevertheless, we found that acute administration of URB597 (0.3 mg kg^−1^, i.p.), which did not alter the time spent in the social chamber or sniffing in wild-type *fmr1^+/+^* (FVB/NJ) mice (Fig. 4a–c), increased the time spent by *fmr1^−/−^* mice in the social chamber and the time spent sniffing to levels to those displayed by control mice (Fig. 4a–c). These results suggest that the prosocial action of increased anandamide activity (through FAAH inhibition) is generalizable across at least two distinct models of ASD-related social impairment, without affecting socially normal animals. We did not test *fmr^−/−^* mice in the elevated plus maze test because these mice display an innate preference for the open arms of the elevated plus maze,^28^ which might confound data interpretation.

## Discussion

For better or worse, desperate parents are treating their autistic children with various forms of marijuana. Successes have been reported in high-profile anecdotes publicized by social media. This societal experiment highlights the lack of scientific knowledge regarding the therapeutic utility and safety of cannabinoid agents in ASDs. The present study provides an initial test of the idea that enhancing anandamide-mediated endocannabinoid signaling might help to alleviate social impairment in ASD. We show that inhibition of the anandamide-deactivating enzyme FAAH corrects social impairment in two distinct ASD-related models—BTBR and *fmr1^−/−^* mice. We confirm that FAAH inhibition is an appropriate strategy to elevate levels of anandamide, and thus, anandamide signaling, in BTBR mice. Furthermore, we show that the prosocial action of FAAH inhibition is independent of reducing anxiety in BTBR mice. Together, the results put forth FAAH as a novel therapeutic target for ASD-related social impairment.

Separate lines of previous research suggest the general notion that abnormal endocannabinoid signaling might contribute to ASD. First, endocannabinoids play important roles in neurodevelopment, which is also affected by exogenous cannabinoids.^33^ Second, ASD-related alterations in synaptic signaling have been linked to the endocannabinoid system. For example, mutations in neuroligins, a family of ASD-linked synaptic adhesion proteins, impair tonic endocannabinoid signaling.^34^ In a related example, we found that deletion of FMRP, the mRNA-trafficking protein missing in Fragile X Syndrome, impairs the formation of a key signaling complex that links metabotropic glutamate receptor-5 (mGluR5) and the 2-AG-synthesizing enzyme diacylglycerol lipase-α (DGL-α).^28^ Third, ASD-related insults disturb resting endocannabinoid levels or endocannabinoid system components. For instance, we found that chronic isolation increases 2-AG in the prefrontal cortex, without affecting anandamide, and increases both 2-AG and anandamide in the piriform cortex.^35^ Furthermore, developmental treatment with valproic acid reduces cerebellar mRNA levels of DGL-α and reduces hippocampal mRNA levels of the 2-AG-hydrolyzing enzyme monoacylglycerol lipase.^36^ Importantly, however, these lines of research have not addressed whether deficient endocannabinoid signaling contributes to the core component of ASD—social impairment.

A limited literature has hinted at this possibility indirectly by suggesting a role for endocannabinoid signaling in normal social behaviors. Genetic removal of CB_1_ receptors alters social interactions in mice in a context-dependent manner,^32^ which may be related to social anxiety and/or cognition.^37,38^ CB_1_ agonists impair social play in rats.^39^ In contrast, genetic removal of FAAH in mice increases social interactions,^13^ and FAAH inhibition promotes social play in rats.^14^ Thus, the bidirectional modulation of social behavior likely depends on the dose and the identity of the affected circuits. We recently identified a signaling mechanism in male mice by which oxytocin drives anandamide-mediated endocannabinoid signaling to control social reward.^15^ In addition, human studies have found that marijuana may enhance sociability^16,17^ and a polymorphism in the CB_1_ cannabinoid receptor gene modulates social gaze.^18^

Given that endocannabinoid signaling has been linked to ASD and might play a role in normal social behavior, we focused the present investigation on the possible role of endocannabinoid signaling in the social impairment component of ASD. We found that that social impairment is corrected in two distinct mouse models by increasing anandamide activity through FAAH inhibition.

This correction raises the immediate question of whether increasing anandamide activity in these mice is prosocial *per se* or simply anxiolytic. This question is particularly important in light of the known roles of endocannabinoids in stress modulation.^19,30,40,41^ In our case, we found that the prosocial action of anandamide is unlikely to be due to general anxiolysis, because FAAH inhibition did not alter BTBR performance in the elevated plus maze when tested in the dim lighting conditions of the social approach test. This result is in line with previous reports showing that FAAH inhibition results in anxiolytic-like effects only under “high-light” conditions.^42^ While this phenotype does not exclude more nuanced forms of social anxiety, it does support the idea that the modulation of stress reactivity and social behavior can be dissociable. In line with this notion, CB_1_ overexpression in the medial prefrontal cortex alters social interactions without overtly changing the anxiety-related phenotype.^43^

In contrast to socially impaired mice, parallel experiments in normal mice indicated that increasing anandamide does not alter social approach. One explanation could be technical—namely, that the social approach test has a ceiling effect or is unable to capture more subtle qualities of social interaction.^44^ Indeed, the task is typically used as a screening tool rather than a continuous scale measure of sociability. Another explanation could be biological—that signaling systems in a healthy brain are able to compensate for endocannabinoid enhancement in a way that socially impaired brains cannot. These explanations are in line with previous reports of different social situations in which *faah^−/−^* mice demonstrated increased direct reciprocal interactions,^13^ as well as URB597-treated juvenile rats engaging in more social play.^14^ Therefore, expanded investigation is warranted into how anandamide contributes to different social contexts and the qualities of social interactions. Nevertheless, our set of results suggests that the prosocial action of FAAH blockade is selective for social impairment in certain contexts, which may be therapeutically advantageous for the spectral nature of ASD.

Based on our results and the available literature, we can reasonably speculate on two possible scenarios improved by anandamide signaling that may underlie social impairment in BTBR and *fmr1^−/−^* mice. First, oxytocin-driven anandamide activity in the NAc, which we previously demonstrated to be important for social reward,^15^ may be impaired in these mice. Consistent with this idea, BTBR mice were found to have abnormal oxytocin expression in the hypothalamus.^45^ BTBR mice were also reported to be deficient in conditioned place preference to social interactions.^46^ However, because social conditioned place preference is a relatively new construct, and the learning impairments in these mice make interpretation problematic, further support from the literature is lacking. A second possible scenario is that anandamide might correct an imbalance of excitatory and inhibitory neurotransmission in the cortex, which has been postulated to underlie ASD.^47^ Enhancing GABAergic activity in BTBR mice ameliorates their social impairment, and negative allosteric GABA modulation in C57Bl6J mice recapitulates social impairment.^48^ This suggests that a loss of balance between inhibitory and excitatory activity might contribute to social impairment. A simplified view of this result orients us to interpret our findings as indicating that anandamide could modulate such balance. This view is consistent with the presence of CB_1_ receptors on presynaptic terminals of both glutamatergic projection neurons and GABAergic interneurons.^11^

In conclusion, the present study provides new insights into the role of endocannabinoid signaling in social behavior and validates FAAH as a novel therapeutic target for the social impairment of ASD.

## Abbreviations Used

ANOVA: analysis of variance
ASD: autism spectrum disorder
DGL-α: diacylglycerol lipase-α
FAAH: fatty acid amide hydrolase
H^2^-OEA: H^2^-oleoylethanolamide
^2^H_8_-2-AG: ^2^H_8_-2-arachidonoyl-*sn*-glycerol
LC/MS: liquid chromatography–mass spectrometry
mGluR5: metabotropic glutamate receptor-5
NAc: nucleus accumbens
Rt: retention times
